# Supplementary material for: Tolerance to ambiguous uncertainty predicts prosocial behavior
Source: Nat Commun. 2018 Jun 12;9:2156. doi: 10.1038/s41467-018-04631-9 (PMC5997641; doi:10.1038/s41467-018-04631-9)
Supplement: Supplementary file 1 — Supplementary Information [file 41467_2018_4631_MOESM1_ESM.pdf]

SUPPLEMENTARY INFORMATION

Tolerance to ambiguous uncertainty predicts prosocial behavior

SUPPLEMENTARY METHODS

Gambling Task

Supplementary Table 1

RISK	*AMBIGUITY*
Risk 25% $5	Ambiguity 24% $5
Risk 25% $8	Ambiguity 24% $8
Risk 25% $20	Ambiguity 24% $20
Risk 25% $50	Ambiguity 24% $50
Risk 25% $125	Ambiguity 24% $125
Risk 50% $5	Ambiguity 50% $5
Risk 50% $8	Ambiguity 50% $8
Risk 50% $20	Ambiguity 50% $20
Risk 50% $50	Ambiguity 50% $50
Risk 50% $125	Ambiguity 50% $125
Risk 75% $5	Ambiguity 74% $5
Risk 75% $8	Ambiguity 74% $8
Risk 75% $20	Ambiguity 74% $20
Risk 75% $50	Ambiguity 74% $50
Risk 75% $125	Ambiguity 74% $125

Task: Supplementary Table 1 depicts all the combinations of lotteries subjects were presented with across the entire task. Each trial type was presented twice in two blocks, for a total of 62 trials. The lotteries represented actual bags filled with red and blue chips placed in the testing lab and were used to pay subjects (see below). The colors associated with the winning gamble, and the side in which the lotteries were placed on the screen (left-right) were counterbalanced throughout the task. Because prior results suggest that ambiguity and risk attitudes are stable across the gain and loss domain (e.g. aversion to uncertainty is a constant preference and is greater in the loss than gain domain[1]), we only measured risk and ambiguity within the gain domain. For the online gambling task (Experiment 3), all monetary amounts were divided by a factor of 20 to make it appropriate for Amazon Mechanical Turk. Because there was no physical lab space in the online experiment, there were no actual bags filled with red and blue chips. All other aspects of the online gambling task were the same.

Detailed instructions: Before beginning the task, subjects were told that all the decisions they made during the gambling task could influence their monetary payoff. They were explicitly instructed that: "*We have multiple bags lined up. Each bag has a picture that matches each lottery image used in the task. The probability of each bag is stated in accordance to the number of blue and red chips it contains. These are the same probabilities you will see during the task on*

the computer. Your choices during the task will determine your payment. At the end of the task, one trial will be randomly picked: if on that trial, you chose the sure outcome of $5, you will receive the $5, if you chose the lottery, we will play the lottery using the bags you see here in the room. You will draw a chip from inside the bag and you will get the payoff accordingly to what was stated in the lottery. After the lottery, you will be allowed to look inside the bags to see that they match the stated probability or ambiguity level pictured here."

Spanish Sample for Experiment 1

Procedure: For the subjects run at Universitat Pompeu Fabra, all materials were translated to Spanish to avoid any possible effect of language[2]. Subjects played in groups of 15-20 in individualized computer cubicles and were not allowed to interact during the task. Subjects first completed the Gambling task and then the Public Goods Game. Bags representing each gamble were present in the testing room during the session. Before beginning each task, subjects carefully read the instructions and answered a comprehensive set of questions to ensure proper understanding. If subjects failed to respond correctly to any of the comprehension questions, the researcher clarified the task parameters before continuing. Two participants were randomly chosen in each session to be paid out from one realized trial from the Gambling task and one realized trial from the Public Goods Game.

Trust Game

Stimuli: We used 12 color images of non-smiling white male faces taken them from the UTEP database. These 12 images were selected based on ratings (i.e., 'Attractiveness', 'Trustworthiness' and 'Overall Positive or Negative Feeling') from a separate behavioral cohort (N=24). To rate each face, subjects used a sliding bar ranging from 1 to 10 (where 1=not at all and 10=very). Faces were selected that fell within 1 standard deviation of the mean for all three of these ratings. These faces were then randomized and matched with a different trustee profile for each participant to ensure there were no demand effects of a given face.

Type of Trustee: In the Trust Game, we created three different types of trustees by manipulating the proportion of money they shared back to the Investor (subjects). The

Trustworthy Trustee typically reciprocated (the maximum money was reciprocated in 4/10 trials, 90% of the maximum amount was reciprocated in 3/10 trials, 80% of the maximum amount was reciprocated in 2/10 trials, and 70% of the maximum amount was reciprocated in 1/10 trials, Fig 2B, Supplementary Table 3). The Untrustworthy Trustee typically kept all the money (no money was reciprocated in 4/10 trials, 10% of the maximum amount was reciprocated in 3/10 trials, 20% of the maximum amount was reciprocated in 2/10 trials, and 30% of the maximum amount was reciprocated in 1/10 trials, Fig 2B). The Neutral Trustee was equally likely to reciprocate as to defect (no money was reciprocated in 1/10 trials, 10% of the maximum amount was reciprocated in 1/10 trials, 20% of the maximum amount was reciprocated in 1/10 trials, and so forth, up to 1/10 trials in which 100% of the maximum amount was reciprocated, which resulted in a flat distribution, Fig 2B). In addition, we also exogenously manipulated the level of ambiguity associated with the trustees by creating two different feedback rates: unambiguous feedback, in which feedback about the partner's actions is always presented, and ambiguous feedback, in which feedback is only presented 50% of the time. Together, this led to six different types of trustees. The order of presentation of type of trustee was randomized across subjects.

Supplementary Table 2: All trial types by each type of trustee.

	TYPE OF TRUSTEE	
TRUSTWORTHY	***NEUTRAL***	***UNTRUSTWORTHY***
Maximum amount of money shared back	Maximum amount of money shared back	No amount of money shared back
Maximum amount of money shared back	90% of maximum amount shared back	No amount of money shared back
Maximum amount of money shared back	80% of maximum amount shared back	No amount of money shared back
Maximum amount of money shared back	70% of maximum amount shared back	No amount of money shared back
90% of maximum amount shared back	60% of maximum amount shared back	10% of maximum amount shared back
90% of maximum amount shared back	40% of maximum amount shared back	10% of maximum amount shared back
90% of maximum amount shared back	30% of maximum amount shared back	10% of maximum amount shared back
80% of maximum amount	20% of maximum	20% of maximum amount

shared back	amount shared back	shared back
80% of maximum amount shared back	10% of maximum amount shared back	20% of maximum amount shared back
70% of maximum amount shared back	No amount money shared back	30% of maximum amount shared back

SUPPLEMENTARY TABLES & FIGURES

Descriptive statistics of model parameters for the Gambling Task: In accordance with past research[3–5], ambiguity (β) and risk (α) attitudes either did not correlate with one another or did so minimally (Experiment 1: R=0.24, p=0.01; Experiment 2: R=0.21, p=0.21; Experiment 3: R=-0.22, p=0.10). Interestingly, the population-level differences (reported below in Supplementary Table 3) between the subjects collected across the two locations for Experiment 1 were significantly different (Supplementary Table 4). While Americans were more ambiguity tolerant than Spaniards (t(101)=-2.61, p=0.01; Fig. S1), risk attitudes did not differ between the two populations (t(101)=0.66, p=0.51).

Supplementary Table 3: Population-level descriptive statistics of model parameters.

		Mean	SD	Min	Max
Experiment 1	Alphas	.61	.23	0	1.47
	Betas	.43	.45	-1.5	1.21
Experiment 2	Alphas	.66	.21	.38	1.18
	Betas	.42	.40	-.59	1.14
Experiment 3	Alphas	.34	.15	.08	0.72
	Betas	-.26	.60	-1.42	1.28

Supplementary Table 4: Population-level descriptive statistics of model parameters for Experiment 1 between the two locations.

		Mean	SD	Min	Max
Americans	Alphas	.63	.30	0	1.47
	Betas	.28	.61	-1.5	1.18
Spaniards	Alphas	.60	.19	.23	1.16
	Betas	.52	.31	-.42	1.21

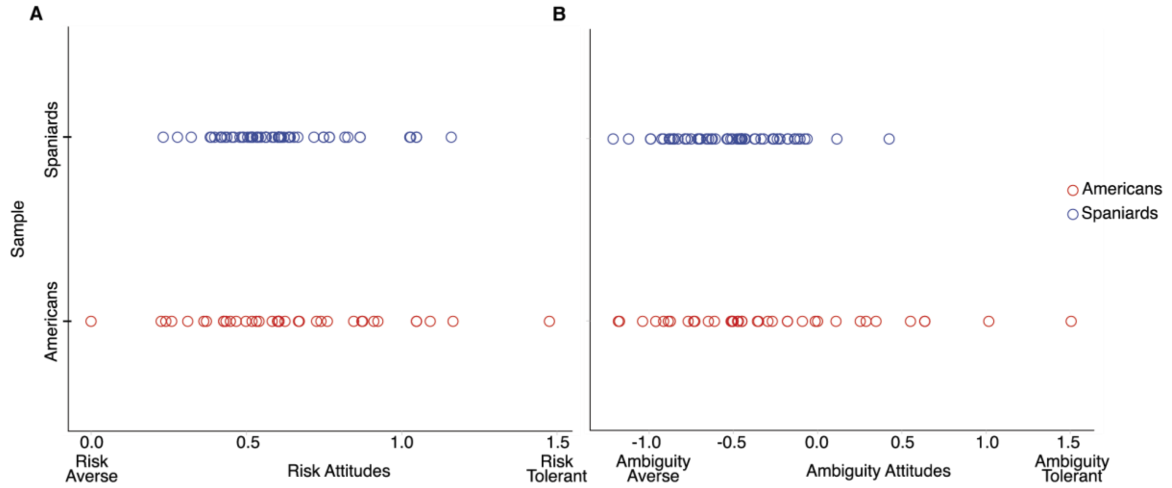

Supplementary Figure 1 | Panel A depicts risk attitudes (α) and panel B depicts ambiguity attitudes (β) for Experiment 1, split by the two samples. The distribution of risk attitudes was similar across the samples. However, there was a significant difference in ambiguity attitudes between the two samples, whereby Americans were more ambiguity tolerant than Spaniards, $p<0.01$. Ambiguity attitudes are inverted to align with Risk attitudes.

Experiment 1

Cooperative behavior as a function of ambiguity and risk attitudes modeled separately. A hierarchal linear regression modeling cooperative behavior as a function of ambiguity attitudes and trial number (Supplementary Table 5), and another as a function of risk attitudes and trial number (Supplementary Table 6) were run to compare coefficients against one another (the complete model is reported in the manuscript). We found that the coefficient from the simple regression for ambiguity was significantly different from coefficient from the simple regression for risk ($z(102)=2.14$, $p=0.03$).

Supplementary Table 5 | Experiment 1: Ambiguity

$$\text{Cooperation}_{i,t} = \beta_0 + \beta_1 \text{Ambiguity Attitude}_i \times \beta_2 \text{ Trial Number}_{i,t} + \varepsilon$$

DV	*Coefficient* ($\boldsymbol{\beta}$)	*Estimate (SE)*	*t-value*	*P value*
Cooperation	Intercept	-0.15 (.19)	-0.80	0.42
	Trial	-0.08 (.01)	-6.44	<0.001***
	Ambiguity Attitude	0.66 (.21)	3.15	0.01**
	Ambiguity Attitude X Trial	-0.03 (.01)	-1.86	0.06†

Note. Where Ambiguity Attitude is indexed by subject (i) and Trial Number is indexed by subject and trial (i,t). Ambiguity attitude were inverted to align on the same scale as risk attitudes and risk and ambiguity attitudes were

standardized before being entered into the regression. Cooperation is coded as defect (0) and cooperate (1). † p<0.1, **p<0.01, ***p<0.001. AIC=2013.2.

Supplementary Table 6 | Experiment 1: Risk

$$\text{Cooperation}_{i,t} = \beta_0 + \beta_1 \text{Risk Attitude}_i \times \beta_2 \text{ Trial Number}_{i,t} + \varepsilon$$

DV	*Coefficient* (β)	*Estimate (SE)*	*t-value*	*P value*
Cooperation	Intercept	-0.17 (.19)	-0.86	0.38
	Risk Attitude	0.04 (.20)	0.19	0.85
	Trial	-0.08 (.01)	-6.25	<0.001***
	Risk Attitude X Trial	-0.02 (.01)	-1.23	0.22

Note. Where Risk Attitude is indexed by subject (i) and Trial Number is indexed by subject and trial (i, t). Risk attitudes were standardized before being entered into the regression. Cooperation is coded as defect (0) and cooperate (1). ***p<0.001. AIC=2072.1.

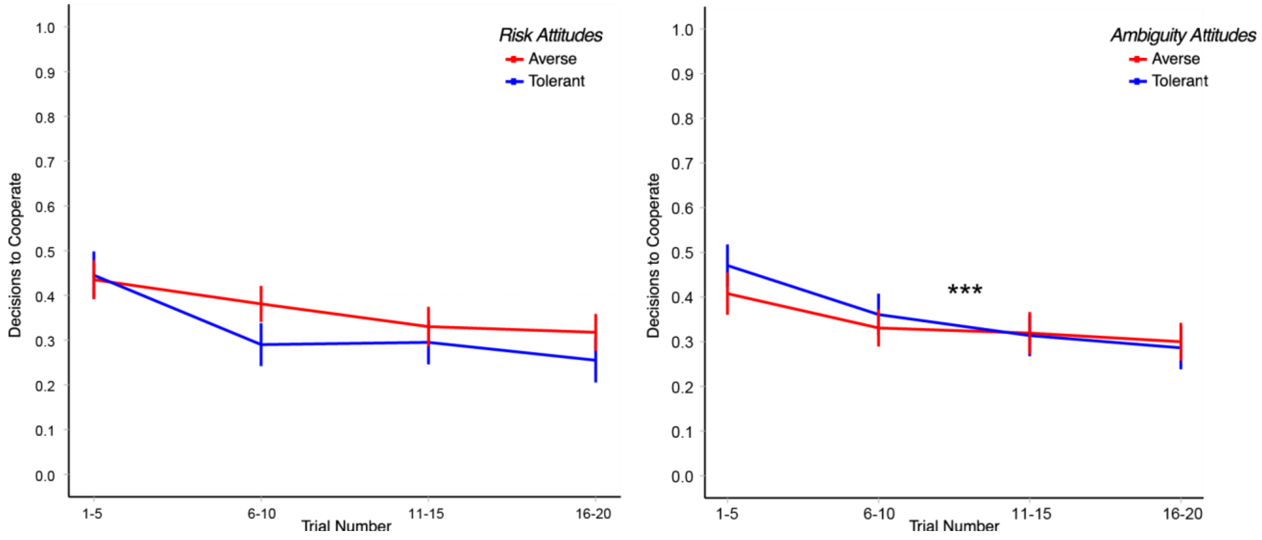

Supplementary Figure 2 | Raw behavioral data from Experiment 1. Risk and ambiguity attitudes are median split for presentation purposes. Subjects' responses are averaged every five trials. Error bars represent ±1 Standard Error of the Mean. Asterisks indicate significant differences (***p <0.001).

Experiment 2

Trusting behavior as a function of risk attitudes: A hierarchal linear regression modeling trusting behavior as a function of risk attitudes and type of trustee revealed that similar to Experiment 1, risk attitudes do not modulate prosocial behavior (Supplementary Table 7). In the same vein, risk attitudes did not show an interactive effect with trustee type. We also ran the complete model incorporating both risk and ambiguity attitudes. As in the simple regressions, there was a relationship between trust and ambiguity tolerance (Supplementary Table 8). The

direct comparison between the coefficients for the interaction between Untrustworthy Trustee and risk and ambiguity attitudes was also significant (z(36)=1.98, p=0.04). Together, this provides converging evidence that risk attitudes are not a relevant psychological mechanism driving prosocial decisions.

Supplementary Table 7 | Experiment 2: Risk

$\text{Money Trusted}_{i,t} = \beta_0 + \beta_1 \text{Risk Attitude}_i \times \beta_2 \text{ Type Of Trustee}_{i,t} + \varepsilon$

DV	*Coefficient* ($\boldsymbol{\beta}$)	*Estimate (SE)*	*t-value*	*P value*
Money Trusted	Intercept	0.91 (.11)	8.54	<0.001***
	Risk Attitude	0.10 (.10)	0.98	0.33
	Untrustworthy	-0.26 (.06)	-4.28	<0.001***
	Trustworthy	0.63 (.09)	6.87	<0.001***
	Risk Attitude x Untrustworthy	-0.08 (.06)	-1.26	0.21
	Risk Attitude x Trustworthy	-0.03 (.09)	-0.37	0.71

Note. Risk Attitude is indexed by subject (i) and Type of Trustee is a categorical variable such that the neutral Trustee serves as the reference category. Risk attitudes were standardized before being entered into the regression. ***p<0.001. AIC=10,494

Supplementary Table 8 | Experiment 2: Risk and Ambiguity

$\text{Money Trusted}_{i,t} = \beta_0 + \beta_1 \text{Risk Attitude}_i \times \beta_2 \text{ Type Of Trustee}_{i,t} + \beta_3 \text{Ambiguity Attitude}_i \times \beta_4 \text{ Type Of Trustee}_{i,t} + \varepsilon$

DV	*Coefficient* ($\boldsymbol{\beta}$)	*Estimate (SE)*	*t-value*	*P value*
Money Trusted	Intercept	0.91 (.11)	8.55	<0.001***
	Risk Attitude	0.10 (.11)	0.91	0.36
	Ambiguity Attitude	-0.01 (.11)	-0.11	0.91
	Untrustworthy	-0.26 (.06)	-4.50	<0.001***
	Trustworthy	0.63 (.09)	6.94	<0.001***
	Risk Attitude x Untrustworthy	-0.05 (.06)	-0.76	0.44
	Risk Attitude x Trustworthy	-0.01 (.09)	-0.15	0.87
	Ambiguity Attitude x Untrustworthy	0.12 (.06)	2.06	0.04*
	Ambiguity Attitude x Trustworthy	0.09 (.09)	0.91	0.36

Note. Where Risk and Ambiguity Attitudes are indexed by subject (i) and Type of Trustee is a categorical variable such that the neutral Trustee serves as the reference category. Ambiguity attitude were inverted to align on the same scale as risk attitudes and risk and ambiguity attitudes were standardized before being entered into the regression. *p<0.05, ***p<0.001. AIC=10,496

Experiment 3

Cooperative behavior in the Prisoner's Dilemma as a function of risk attitudes: To assess the effect of risk attitudes in Experiment 3, we ran a trial-by-trial regression with Risk Attitudes and Type of Player on decisions to cooperate in the sampling phase of the Prisoner Dilemma. We removed two subjects because they had risk attitudes that were more than three standard

deviations above from the mean. As with Experiment 1 and 2, risk attitudes did not influence the probability of cooperation (Supplementary Table 9). We did, however, find an interaction with player type, such that those who were more risk tolerant cooperated more with the cooperative player. This might be due to the nature of the sampling task, which converts a socially ambiguous scenario into one where probabilities can be computed. Only in this specific context, in which past behavior is unambiguously traceable through sampling partners' previous choices, did we find that risk attitudes have a relationship with prosocial behavior.

Supplementary Table 9 | Experiment 3: Prisoner Dilemma after sampling

$$\text{Cooperation}_{i,t} = \beta_0 + \beta_1 \text{Risk Attitudes}_i \times \beta_2 \text{ Type Of Player}_{i,t} + \varepsilon$$

DV	*Coefficient* ($\boldsymbol{\beta}$)	*Estimate (SE)*	*t-value*	*P value*
Cooperation	Intercept	0.54 (.39)	1.40	0.16
	Risk Attitudes	-0.25 (.39)	-0.63	0.52
	Defection Player	-0.77 (.43)	-1.75	0.08†
	Cooperative Player	1.11 (.51)	2.16	0.03*
	Risk Attitudes x Defection Player	-0.05 (.44)	-0.12	0.90
	Risk Attitudes x Cooperative Player	1.78 (.53)	2.94	0.003**

Note. Where Risk Attitude is indexed by subject (i) and Type of Player is a categorical variable. Risk attitudes were standardized before being entered into the regression. Cooperation is coded as defection (0) and cooperation (1). † p<0.1, *p<0.05, **p<0.01. AIC=221.98

Supplementary References

1. Tymula, A. *et al.* Adolescents' risk-taking behavior is driven by tolerance to ambiguity. *Proc. Natl. Acad. Sci. U. S. A.* **109,** 17135–40 (2012).
2. Costa, A., Foucart, A., Arnon, I., Aparici, M. & Apesteguia, J. 'Piensa' twice: On the foreign language effect in decision making. *Cognition* **130,** 236–254 (2014).
3. Levy, I., Snell, J., Nelson, A. J., Rustichini, A. & Glimcher, P. W. Neural representation of subjective value under risk and ambiguity. *J. Neurophysiol.* **103,** 1036–1047 (2010).
4. FeldmanHall, O., Glimcher, P., Baker, A. L. & Phelps, E. A. Emotion and decision-making under uncertainty: Physiological arousal predicts increased gambling during ambiguity but not risk. *J. Exp. Psychol. Gen.* **145,** 1255–1262 (2016).
5. Tymula, A., Rosenberg Belmaker, L. A., Ruderman, L., Glimcher, P. W. & Levy, I. Like cognitive function, decision making across the life span shows profound age-related changes. *Proc. Natl. Acad. Sci.* **110,** 17143–17148 (2013).
